# 上调*Delta-Like1*基因可增强小细胞肺癌化疗敏感性

**DOI:** 10.3779/j.issn.1009-3419.2013.06.02

**Published:** 2013-06-20

**Authors:** 换新 刘, 娟 彭, 义凤 白, 琳琅 郭

**Affiliations:** 510282 广州，南方医科大学附属珠江医院病理科 Department of Pathology, Zhujiang Hospital, Southern Medical University, Guangzhou 510282, China

**Keywords:** DLL1, 多药耐药, 肺肿瘤, Delta-Like1 (DLL1), Mul-tidrug resistance, Lung neoplasms

## Abstract

**背景与目的:**

DLL1（Delta-Like1）与Notch受体结合激活Notch信号通路，从而决定细胞的分化，并调控多种组织的生长发育。已有研究报道DLL1与肿瘤的生长、分化密切相关。前期基因芯片发现DLL1与小细胞肺癌的耐药性相关，本研究旨在进一步探讨DLL1在小细胞肺癌多药耐药中的作用。

**方法:**

首先通过QRT-PCR和Western blot从基因和蛋白水平检测化疗敏感细胞株H69及多药耐药细胞株H69AR中DLL1的差异表达；转染DLL1-pIRES2-EGFP表达质粒上调H69AR细胞中的DLL1的表达，构建稳定转染的过表达细胞株H69AR-eGFP-DLL1，通过CCK8检测细胞对各种化疗药物（ADM, DDP, VP-16）的敏感性变化，流式细胞仪检测细胞周期及凋亡的变化。

**结果:**

DLL1在化疗敏感细胞H69中的表达明显高于H69AR，过表达H69AR中DLL1的表达能够增加细胞对化疗药物的敏感性，促进细胞的凋亡，细胞周期发生G_0_/G_1_期及S期阻滞，上调DLL1增加其下游基因*HES1*、*HEY1*的表达。

**结论:**

在小细胞肺癌中上调DLL1的表达可能增加细胞对化疗药物的敏感性，DLL1通过肿瘤细胞间的相互作用激活*HES1*、*HEY1*等下游基因，影响小细胞肺癌的多药耐药。

## Abstract (en)

**Background and objective:**

Delta-Like1 (DLL1) can combine with Notch receptor and activate the Notch signal pathway, then made a decision to cell differentiation and regulate the development of many tissues. It is proved that DLL1 was highly correlated with tumor'growth and differentiation, our previously study showed that DLL1 was associated with MDR in small cell lung cancer (SCLC). The aim of this study is to furtherly investigate the role of *DLL1* gene in small cell lung multi-drug resistance.

**Methods:**

Firstly, the analysis of qRT-PCR and Western blot were used to study differential expression of DLL1 from mRNA and protein levels in both the H69 and H69AR cell lines. Then, we developed a stably DDL1 overexpressing H69AR-eGFP-DLL1 subline, by transfection with DLL1-pIRES2-EGFP. Moreover, the sensitivities of cells to chemotherapy drugs such as ADM, DDP, VP-16 were detected by CCK8 assay. The change of cell cycle and apoptosis rate were detected by flow cytometry.

**Results:**

The expression of DLL1 was significantly decreased in H69AR cells than that in the H69 cells. The sensitivities of H69AR cells to chemotherapy drugs were increased when up-regulated the expression of DLL1, enforced DLL1 expression increased cell apoptosis and the cell cycle arrest in G_0_/G_1_ and S phase in H69AR cells, the expression of downstream genes *HES1* and *HEY1* were increased after transfected with DLL1-pIRES2-EGFP.

**Conclusion:**

Our results suggest that overexpression of DLL1 in small cell lung cancer may increase the sensitivity of cells to chemotherapeutic agents. DLL1 influence drug resistance of small cell lung cancer through activating transcription of downstream genes *HES1* and *HEY1*.

小细胞肺癌（small cell lung cancer, SCLC）的细胞倍增时间短，病情进展快，早期即发生血道和淋巴道转移，恶性程度在所有肺癌类型中最高。SCLC的治疗以放化疗为主，尽管80%患者早期对放、化疗呈现出较好的初始反应性，但很快即发生复发或病情进展。局限期患者5年生存率低于30%，广泛期患者5年生存率仅1%-2%^[1]^。耐药形成，尤其是多药耐药（multidrug resistance, MDR）的产生是SCLC化疗失败的最重要原因^[2, 3]^。因此，化疗抗药性已成为目前SCLC临床治疗急需解决的问题之一。

DLL1（Delta-Like1）为单次跨膜糖蛋白，属于DSL（Delta, Serrate, Lag-2）蛋白家族，人类*DLL1*基因定位于染色体6q27，全长3.04 kb中，ORF编码723个氨基酸，是脊椎动物Notch的两个配体之一，它与Notch受体结合激活Notch信号通路，决定细胞分化的命运，参与调控许多组织的生长发育。DLL1的细胞内区域与E3泛素连接酶特异结合，使DLL1泛素化和内吞，激活Notch信号通路所必须的结构域^[4-6]^。已有研究^[7, 8]^报道DLL1与肿瘤的生长、分化密切相关，但DLL1对肿瘤耐药方面的研究还很少见，尤其在SCLC耐药中的作用目前国内外还未见相关的报道。我们前期通过基因表达谱芯片对SCLC耐药细胞H69AR和非耐药细胞H69中21, 522个基因进行分析，结果发现H69AR细胞中包含DLL1在内的1, 131个基因表达下调^[9]^，1, 252个基因表达上调，本实验旨在进一步验证DLL1在SCLC敏感细胞株H69和多药耐药株H69AR中的表达，以及其表达对SCLC化疗药物敏感性及细胞周期与凋亡的影响。

## 1 材料与方法

### 1.1 材料

pIRES2-EGFP质粒、感受态细菌为本实验室保存。人SCLC敏感细胞株（H69）和其阿霉素耐药株（H69AR）均购自美国ATCC，新生胎牛血清、RPMI-1640培养基购自美国Gibco公司；顺铂、阿霉素和依托泊苷购自辉瑞公司；CCK8及凋亡检测试剂盒购自上海碧云天公司；第1链cDNA合成试剂盒、聚合酶链反应（PCR）试剂盒、DNA maker、DNA纯化试剂盒、质粒提取试剂盒、反转录试剂盒、限制性内切酶等购自大连宝生生物公司；脂质体Lipofetamine 2000购自Invitrogen公司；兔抗人单克隆抗体DLL1购自美国Santa Cruz公司；羊抗兔二抗购自武汉博士德生物公司。

### 1.2 方法

#### 1.2.1 实时荧光定量PCR分析DLL1及其下游基因的mRNA表达

提取细胞中的总RNA进行逆转录和实时荧光定量PCR。使用SYBR实时定量反应试剂盒（Takara）对实验组和对照组细胞中的*DLL1*基因及其下游基因的表达进行分析。Real-time PCR反应及数据分析在ABI PRESM 7500实时定量反应仪上完成，引物由Takara公司合成。DLL1 Forward：AGGGTGTGATGACCAACATGGA；DLL1 Reverse：ATCGGATGCACTCATCGCAGTA；HES 1 Forward：AAAGACGGCCTCTGAGCAC；HES1 Reverse：GGTGCTTCACAGTCATTTCCA；HEY1 Forward：CATGAAGAGAGCTCACCCAGA；HEY1 Reverse：CGCCGAACTCAAGTTTCC；内参照GAPDH上游引物为5’-GGAAGGACTCATGACCACAGTCC-3’，下游引物为5’-TCGCTGTI'GAAGTCAGAGGAGACC-3’。逆转录反应及PCR参照试剂盒说明，以DLL1及其下游基因的上下游引物进行PCR扩增，PCR反应在实时定量PCR反应仪上进行。3次独立实验后得到的数据运用公式RQ=2^-∆∆Ct^的方法进行分析。

#### 1.2.2 过表达pIRES2-EGFP-DLL1的转染及稳定转染细胞株的筛选

① 转染前1天，胰酶消化H69AR细胞并计数，细胞铺板，加入含20%胎牛血清的RPMI-1640细胞培养液，使其在转染日密度为60%-80%；②在两个无菌的Eppendoff管中，分别将1 μL纯化的质粒pLEGFP-N1-DLL1（浓度1 μg/μL）和1 μL纯化的质粒pLEGFP-N1（浓度1 μg/μL）空载体质粒，各用50 μL的无血清无抗生素的Opti-MEM进行稀释、混匀，制成溶液A和B。在另一个Eppendoff管中，将4 μL Lipofectamine^TM^ 2000用100 μL的无血清无抗生素的Opti-MEM进行稀释、混匀，制成溶液C。在5 min内将A和50 μL C、B和50 μL C混匀，室温静置20 min；③等待期间，将培养板中的H69AR细胞用无血清的RPMI-1640培养液洗涤3次，加入400 μL无血清无抗生素的Opti-MEM培养基；④将AC、BC混合物加于H69AR细胞表面，轻轻来回晃动培养板，使混合物均匀覆盖于细胞表面，37 ℃、5%CO_2_孵育；⑤6 h后吸去培养液，将细胞用新鲜的培养液洗涤2次，加入含20%胎牛血清的无抗生素的RPMI-1640培养基继续培养。转染48 h后荧光显微镜下观察荧光强度，检测转染效率；⑥第2天细胞按1:8传代，正常培养基培养；⑦第3天培养基换成含筛选浓度（400 μg/mL）的G418的10%胎牛血清RPMI-1640培养基进行筛选培养；⑧3周后待形成阳性单细胞克隆群落后，用尖吸管吸取单克隆阳性细胞培养，改用含半浓度G418（200 μg/mL）的培养基扩大培养。

#### 1.2.3 Western blot分析DLL1蛋白表达

提取细胞总蛋白，BCA法蛋白定量，每孔中加样50 μg蛋白，经10%SDS-PAGE后，电转移至PVDF膜。5%BSA/TBST室温封闭1 h，加入兔抗人DLL1单克隆（1:200）孵育，4 ℃过夜。TBST漂洗3次，用HRP标记的羊抗兔IgG（1:5, 000）孵育，室温2 h，TBST漂洗3次，ECL检测，暗室曝光10 s-10 min，显影。

#### 1.2.4 CCK8法检测药物敏感性

参照顺铂（DDP）、足叶乙苷（VP-16）及阿霉素（ADM）3种化疗药物的血浆高峰浓度，在各种转染细胞中分别加入0.01倍、0.1倍、1倍和10倍血浆高峰浓度的化疗药物，每种药物的每一浓度设4个重复孔；阴性对照组：仅加细胞不加药物，设4个重复孔；空白调零组：仅加细胞培养液，设4个重复孔。以每孔3×10^3^个细胞接种于96孔培养板中，每孔加入200 μL培养液；细胞贴壁后，将3种化疗药物按不同浓度加入各孔细胞，继续常规培养24 h；每孔加新鲜配制的CCK8溶液20 μL，37 ℃、5%CO_2_下继续培养0.5 h-4 h后，终止培养。选择450 nm波长，在酶联免疫检测仪上测定各孔光吸收值，取每4个重复孔的光吸收值（*A*值）的平均值，计算各种转染细胞在3种化疗药物不同浓度下的存活率；细胞存活率=（实验组*A*值-空白对照组*A*值）/（阴性对照组*A*值-空白对照组*A*值）×100%。重复实验3次，取平均值，以细胞存活率为纵轴，药物浓度对数为横轴作半对数图，并按作图法求出3种药物的IC_50_值。

#### 1.2.5 细胞凋亡检测

对数生长期的细胞以4×10^5^/孔接种6孔板中；37 ℃培养48 h；收集细胞，PBS洗涤2次；细胞重悬于100 μL含Annexin Ⅴ-FITC和0.5 μg PI的结合缓冲液（10 mM HEPES pH7.4; 0.15 M NaCl; 5 mM KCl; 1 mM MgCl_2_; 1.8 mM CaCl_2_）中；避光室温孵育15 min；加入400 μL结合缓冲液；流式细胞仪分析。

#### 1.2.6 细胞周期检测

取对数生长期的细胞，用0.25%胰蛋白酶和0.02%EDTA消化细胞，PBS洗2次，用75%乙醇冰浴固定24 h，然后用含1%BSA的PBS充分混匀洗涤2次，PI染色后进行流式细胞仪测定并用Cell Quest软件分析各组细胞群体在细胞周期各个时相的分布比例。

### 1.3 统计学方法

运用SPSS 13.0统计软件分析，采用*t*检验或*One-way ANOVA*检验，*P* < 0.05为差异具有统计学意义。

## 2 结果

### 2.1 DLL1在敏感株（H69）和耐药株（H69AR）中的差异表达

如图 1A所示，qRT-PCR结果显示H69AR细胞株中DLL1 mRNA表达较H69细胞降低，差异具有统计学意义（*P*=0.003）。Western blot结果也显示在耐药株H69AR中的DLL1蛋白的表达较敏感株H69明显降低（图 1B，*P* < 0.001）。通过PIRES2-EGFP-DLL1上调H69AR细胞株中DLL1的表达：如图 2所示，H69AR分别转染PIRES2-EGFP-NC（A）及PIRES2-EGFP-DLL1（B）后48 h，通过荧光显微镜观察其转染效率达80%（图 2A，图 2B）。QRT-PCR和Western blot检测转PIRES2-EGFP-DLL1后，DLL1在mRNA和蛋白水平上均增高（图 2C，图 2D，*P*=0.004），差异具有统计学意义。提示过表达DLL1的稳定细胞株H69AR-eGFP-DLL1构建成功。细胞对化疗药物敏感性的变化：如图 3所示，CCK8检测显示H69AR对顺铂（DDP），阿霉素（ADM）及足叶乙苷（VP-16）的IC_50_值较敏感细胞株H69增高，提示H69AR对化疗药物的敏感性降低（图 3A，*P*=0.009）。通过转染PIRES2-EGFP–DLL1上调H69AR细胞株中DLL1的表达后，与对照组（H69AR及H69AR-PIRES2-EGFP-NC）相比细胞对DDP，ADM及VP-16的敏感性明显增加, 差异具有统计学意义（图 3B，*P*=0.016）。

**1:**
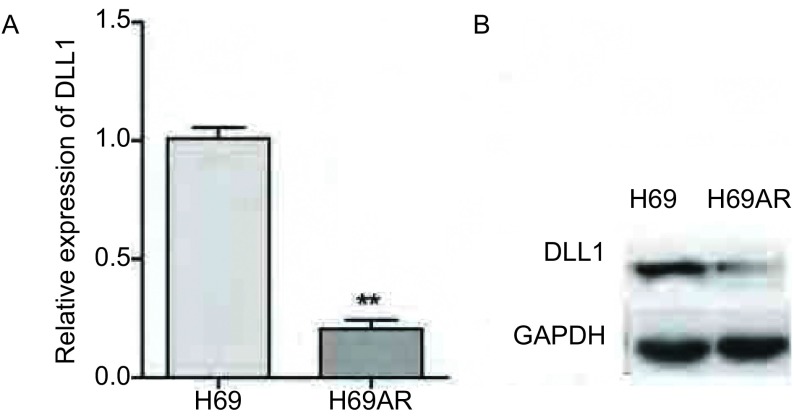
qRT-PCR和Western blot在mRNA水平（A）和蛋白水平（B）检测H69及H69AR细胞中DLL1的表达. ^**^P < 0.01. The expression of DLL1 mRNA (A) and protein (B) levels were assessed by qRT-PCR and Western blot in H69 and H69AR cells. ^**^P < 0.01.

**2:**
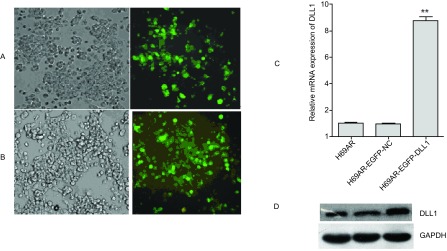
转染PIRES2-EGFP-DLL1过表达质粒上调DLL1的表达。H69AR分别转染PIRES2-EGFP-NC（A）及PIRES2-EGFP-DLL1（B）后48 h，通过荧光显微镜观察其转染效率。细胞转染PIRES2-EGFP-DLL1后，在mRNA水平（C）和蛋白水平（D）检测其对DLL1的表达。光镜，200×（左）；荧光显微镜，200×（右）。^**^P < 0.01. eGFP-NC (negative vector) and eGFP-DLL1 overexpression plasmid were transfected into H69 cells. At 48 h after transfection, uorescent microscopy showed emission green uorescence to detect the transfection efficiency. (A) PIRES2-EGFP-NC; (B) PIRES2-EGFP-DLL1. The expression of DLL1 mRNA (C) and protein (D) after transfected with PIRES2-EGFP-DLL1. Left: Light microscopy, 200×; Right: Fluorescent microscopy, 200×. ^**^P < 0.01.

**3:**
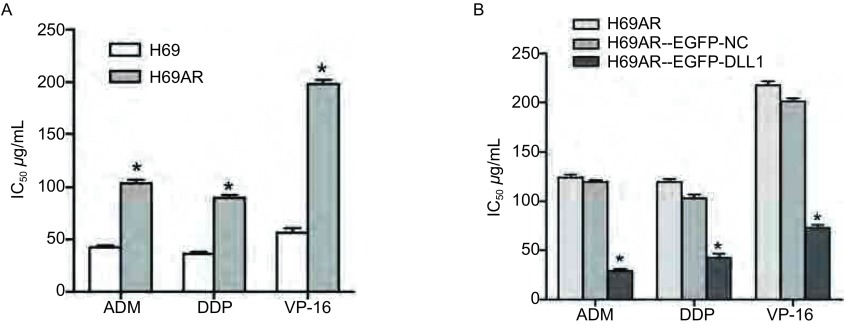
细胞对化疗药物的敏感性的变化。A：CCK8检测H69和H69AR细胞对化疗药物DDP、ADM及VP-16的敏感性；B：通过转染PIRES2-EGFP-DLL1上调H69AR中DLL1的表达后，细胞对DDP、ADM及VP-16的敏感性明显增加。^*^P < 0.05。 The sensitivities of cells to chemotherapy drugs. A: The sensitivities of cells to chemotherapy drugs (ADM, DDP and VP-16) were measured in H69 and H69AR cells; B: The sensitivities of cells to chemotherapy drugs (ADM, DDP and VP-16) were measured after H69AR cells transfected with PIRES2-EGFP-DLL1 or mock by CCK-8 assay. DDP: cis-platinum; ADM: adriamycin; VP-16: etoposide; IC_50_ value: half maximal inhibitory concentration.

### 2.2 细胞凋亡率的变化

如表 1及图 4所示，流式细胞技术检测显示，上调DLL1表达后，H69组凋亡率为（7.294±0.389）%（图 4A），H69AR细胞的凋亡率为（1.954±0.088）%（图 4B），H69AR的凋亡率明显低于H69，两组之间差异具有统计学意义（*P* < 0.001）。而H69AR转染PIRES2-EGFP-DLL1（图 4D）后凋亡率为（17.202±0.872）%较H69AR组及转染PIRES2-EGFP-NC（2.112±0.222）%（图 4C）组明显增高，差异具有统计学意义（*P* < 0.001）。结果提示上调DLL1明显增加H69AR细胞的凋亡。

**1:** 上调DLL1的表达后细胞凋亡率的变化（%, Mean±SD, *n*=5） The apoptosis rate of cells was assayed after transfected with eGFP-DLL1 or a negative control (NC) (%, Mean±SD, *n*=5)

Group	*n*	Apoptosis rate	*F*	*P*
eGFP-DLL1	5	17.202±0.872^▲^	1056.897	< 0.001
NC	5	2.112±0.222		
H69AR	5	1.954±0.088^#^		
H69	5	7.294±0.389		
^#^Compare with H69 group，The difference has stastistical significance, *P* < 0.001; ^▲^Compare with random group，The difference has stastistical significance, *P* < 0.001.				

**4:**
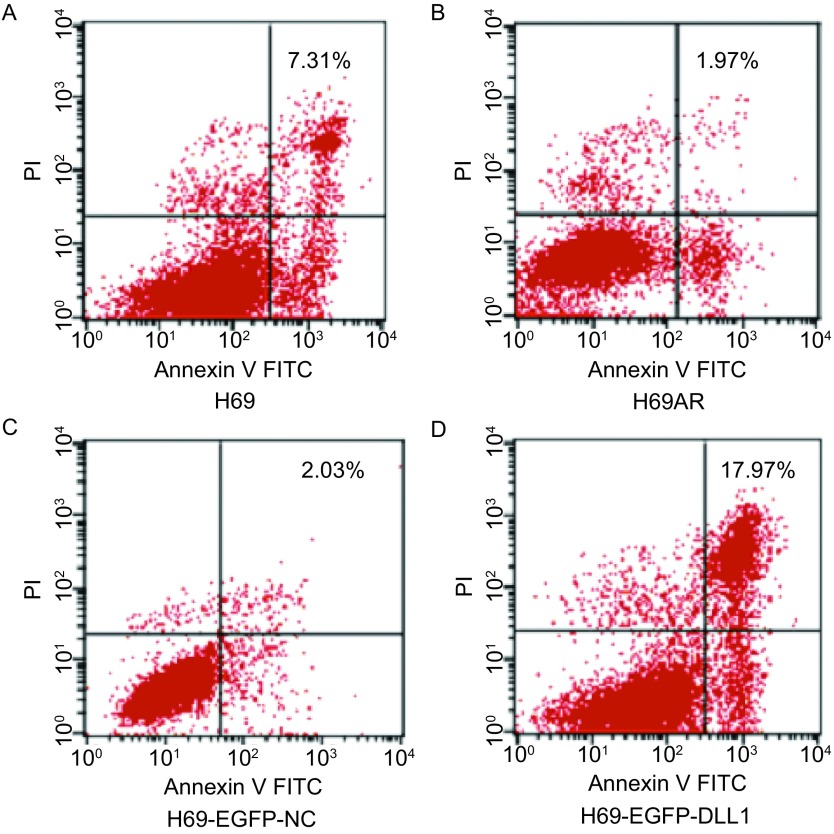
上调DLL1的表达后流式细胞术检测细胞凋亡的变化。A：H69；B：H69AR；C：H69AR-EGFP-NC；D：H69AR-EGFP-DLL1。 Cell apoptosis was assayed by flow cytometric analysis after transfected with eGFP-DLL1 or a negative control (NC). A: H69; B: H69AR; C: H69AR-EGFP-NC; D: H69AR-EGFP-DLL1.

### 2.3 细胞周期的变化

流式细胞技术检测显示，H69组细胞周期主要以G_0_/G_1_期为主（图 5A），H69AR细胞G_2_/M期细胞增多（图 5B），H69AR的G_2_/M期细胞明显较H69细胞增多，两组之间差异具有统计学意义（*P* < 0.01）。而H69AR转染PIRES2-EGFP-DLL1（图 5D）后细胞周期G_0_/G_1_期及S期细胞较H69AR组及转染PIRES2-EGFP-NC（图 5C）组明显增高（*P* < 0.001）。结果提示上调DLL1使细胞周期发生G_0_/G_1_期和S期阻滞（表 2）。

**5:**
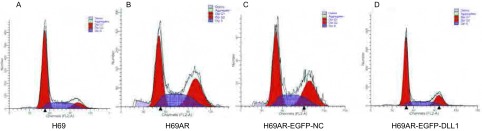
上调DLL1的表达后流式细胞术检测细胞周期的变化。A：H69；B：H69AR；C：H69AR-EGFP-NC；D：H69AR-EGFP-DLL1。 Cell cycles were assayed by flow cytometric analysis after transfected with eGFP-DLL1 or a negative control (NC). A: H69; B: H69AR; C: H69AR-EGFP-NC; D: H69AR-EGFP-DLL1.

**2:** 过表达DLL1后细胞周期分布的百分数（%，Mean±SD，*n*=3） The cell cycles distribution were detected after transfected with eGFP-DLL1 or a negative control (NC) (%, Mean±SD, *n*=3)

Cell cycle	*n*	Cell cycles distribution（%）				*F*	*P*
		eGFP-DLL1	NC	H69AR	H69		
G_0_-G_1_	3	46.272±0.802^▲^	22.604±0.441	23.484±0.544	66.27±0.802	2, 938.186	< 0.001
G_2_-M	3	23.076±0.425^#^	57.021±0.112	58.757±0.155	23.076±0.425	1, 213.429	< 0.001
S	3	29.639±0.381^▲^	20.476±0.472	18.697±0.169	9.639±0.381	1, 490.432	< 0.001
^#^Compare with H69 group，The difference has stastistical significance, *P* < 0.001; ^▲^Compare with random group，The difference has stastistical significance, *P* < 0.001.							

### 2.4 DLL1对下游靶基因的激活

为进一步研究DLL1影响小细胞肺癌耐药的分子机制，我们通过qRT-PCR从基因水平检测了*DLL1*下游基因的表达，如图 6所示，上调*DLL1*的表达，下游靶基因*HES1*及*HEY1*的表达升高，提示DLL1对下游靶基因*HES1*及*HEY1*有激活作用。DLL1下游基因*HES1*、*HEY1*的激活可能是通过肿瘤细胞之间的受体——配体相互作用的结果。

**6:**
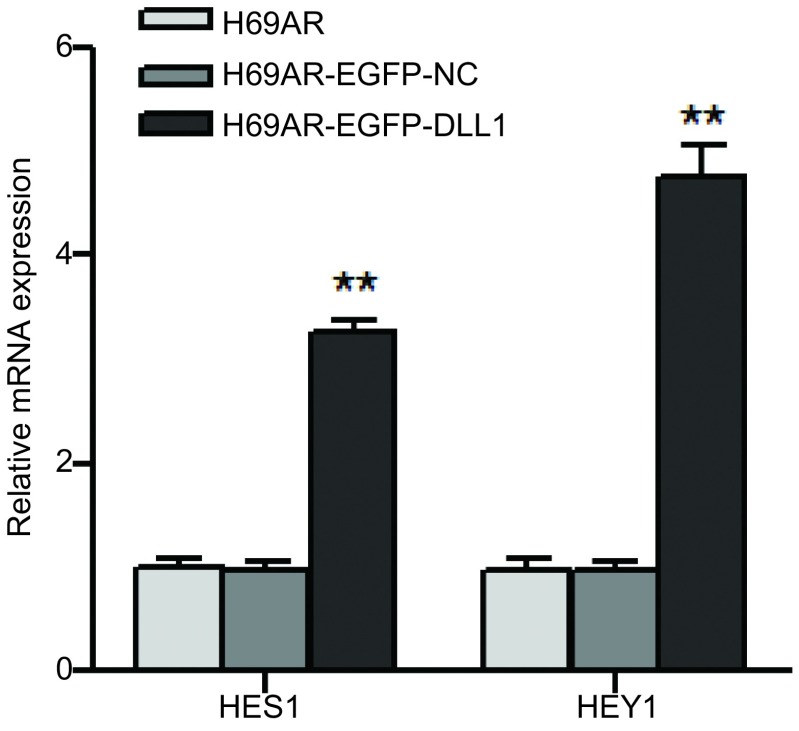
qRT-PCR检测DLL1下游基因的表达 The expression of DLL1 downstream genes *HES1* and *HEY1* were detected by qRT-PCR

## 3 讨论

Notch信号通路是进化上高度保守的细胞与细胞间的信号传导系统，与细胞增殖、分化及凋亡密切相关^[10, 11]^，在胚胎正常发育、机体稳态调控以及成体干细胞的维持中发挥重要作用，该通路的异常激活不仅直接参与肿瘤的发生发展，还与肿瘤耐药密切相关^[12-14]^。近年来的研究^[15-17]^发现Notch-1广泛表达于多种肿瘤细胞，通过促进上皮间质转换（epithelial-mesen chymal transition, EMT）、肿瘤干细胞（cancer stem cells, CSC）表型的改变和调节微小RNA（microRNAs, miRNA）等途径，导致肿瘤对多种化疗药物产生抗药性。因此，Notch-1是对抗肿瘤耐药的潜在靶点。还有研究^[18-23]^表明Notch1受体及其配体delta-like-1（DLL1）在肿瘤的生长、分化、增殖及凋亡中发挥着重要的作用。目前共发现了5种人的Notch配体，分别是DLL1、Delta-like-3（DLL3）、Delta-like-4（DLL4）、JAG1和JAG2。DLL1为单次跨膜糖蛋白，属于DSL（Delta, Serrate, Lag-2）蛋白家族成员，作为Notch信号转导通路的配体之一，目前已有相关研究^[17, 18]^报道DLL1能够抑制肿瘤细胞的增殖和促进细胞分化。人类*DLL1*基因定位于染色体6q27，长度为3.04 kb，其ORF编码723个氨基酸，它与Notch受体结合激活Notch信号通路，决定细胞分化的最终归宿，并参与调控许多组织的生长发育^[23, 24]^。DLL1的细胞内区域与E3泛素连接酶特异结合，该过程称为DLL1泛素化和内吞，此为激活Notch信号通路所必须的结构域^[4-6]^。Notch信号通路正是通过这一机制调控细胞的分化、增殖及凋亡等过程。研究^[21]^报道，MiR-34a通过靶向作用于Notch的配体DLL1损害CD15^+^/CD133^+^肿瘤增殖细胞从而促进髓母细胞瘤的分化。Huang等^[7]^发现选择性的刺激DLL1-Notch信号通路能够恢复T细胞的功能，抑制肿瘤的生长。还有研究^[8]^发现在B16黑色素瘤细胞中上调Notch配体DLL1的表达引起肿瘤血管的减少而抑制肿瘤的生长。

尽管DLL1与肿瘤的生长及分化方面的研究较多，然而该基因及其编码的蛋白质与肿瘤耐药的关系报道很少，与SCLC的多药耐药的相关性目前国内外尚未见相关报道。本实验在前期对SCLC耐药细胞株和敏感细胞株高通量芯片筛选中发现，H69AR耐药细胞株中DLL1的表达较敏感细胞株H69明显降低^[9]^，为了进一步验证芯片结果，我们运用qRT-PCR和Western blot方法进一步从基因和蛋白水平检测了SCLC中DLL1的表达，结果和基因芯片的表达一致。同时，我们还发现，在H69AR细胞株中转染PIRES2-EGFP-DLL1上调DLL1的表达后，肿瘤细胞对化疗药物的敏感性明显增加，流式细胞仪检测显示上调DLL1的表达后细胞凋亡明显增加，细胞周期阻滞在G_0_/G_1_期，提示DLL1与SCLC的耐药相关，上调*DLL1*基因的表达可以提高SCLC耐药细胞株的化疗敏感性，DLL1有可能成为治疗SCLC的靶标。但其具体的机制尚有待于进一步研究。
